# Polymorphism of fibrillar structures depending on the size of assembled Aβ_17-42_ peptides

**DOI:** 10.1038/srep38196

**Published:** 2016-11-30

**Authors:** Mookyung Cheon, Mooseok Kang, Iksoo Chang

**Affiliations:** 1 Center for Proteome Biophysics, Department of Brain and Cognitive Sciences, Daegu Gyeongbuk Institute of Science and Technology (DGIST), Daegu 42988, Korea; 2 Department of Neural Development and Disease, Korea Brain Research Institute, Daegu 41068, Korea

## Abstract

The size of assembled Aβ_17-42_ peptides can determine polymorphism during oligomerization and fibrillization, but the mechanism of this effect is unknown. Starting from separate random monomers, various fibrillar oligomers with distinct structural characteristics were identified using discontinuous molecular dynamics simulations based on a coarse-grained protein model. From the structures observed in the simulations, two characteristic oligomer sizes emerged, trimer and paranuclei, which generated distinct structural patterns during fibrillization. A majority of the simulations for trimers and tetramers formed non-fibrillar oligomers, which primarily progress to off-pathway oligomers. Pentamers and hexamers were significantly converted into U-shape fibrillar structures, meaning that these oligomers, called paranuclei, might be potent on-pathway intermediates in fibril formation. Fibrillar oligomers larger than hexamers generated substantial polymorphism in which hybrid structures were readily formed and homogeneous fibrillar structures appeared infrequently.

Soluble amyloid beta protein (Aβ) oligomers play a pivotal role in the critical neurotoxicity observed in Alzheimer’s disease (AD)^1^,^2^,^3^,^4^. According to the revised amyloid cascade hypothesis, the Aβ oligomers induce an initial toxic effect through potent mechanisms such as direct binding to synaptic receptors, creating a membrane pore or causing unspecific membrane permeabilization^2^,^3^,^4^. Diverse synthetic *in vitro* oligomers as well as brain- or mouse-derived *in vivo* oligomers are suggested to be a toxic species^5^,^6^,^7^,^8^,^9^. However, the list of intermediates or stable oligomers identified in numerous experiments exhibits vast diversity in size and structure^3^. The size of listed oligomers vary from dimers to more than 150-mers displaying prefibrillar or fibrillar structural features, which have been proposed to be on-pathway intermediates for fibril formation or off-pathway stable oligomers^3^. Various oligomers have been identified under heterogeneous experimental conditions, and the molecular details of oligomer structure are not consistent across conditions. Recently, experiments using biased controlled conditions suggested diverse Aβ oligomer structures, such as a stabilized β-hairpin^10^, a dimeric preglobuomer^11^, a β-hairpin trimer^12^, a disk-shaped pentamer^13^, a hexameric barrel^14^, staggered β-sheets^15^, and antiparallel prefibrillar oligomers^16^. However, incoherent results using different experimental conditions hinder our ability to identify a widely acceptable model for endogenous oligomer formation in the human brain. Even oligomers of similar sizes might have different conformations recognized by different antibodies^17^. Therefore, oligomer polymorphism hinders us from focusing on the structural origin of the general toxicity^3^ and is an important factor for identifying the toxic mechanism and developing therapeutic agents.

The majority of experimental studies have tried to detect oligomer structure, which usually generate stable off-pathway or long-lived species^11^,^12^,^13^,^14^,^15^,^16^. For on-pathway intermediates, a few structural features, such as parallel β-sheets or β-hairpins, have been proposed^10^,^18^. However, a general structural model of on-pathway oligomers has been difficult to capture owing to their transient meta-stability and different kinetic pathways^1^. One of the well-known on-pathway mechanisms is the nucleation-dependent polymerization (NP) pathway, in which a nucleus is formed, the process of monomer addition dominates fibril growth, and the template oligomer is believed to be in fibrillar form^19^. Another known on-pathway is the nucleated conformational conversion (NCC) pathway, in which a disordered oligomer is converted to an ordered oligomer^20^. Because these different kinetic pathways depend on experimental conditions, it has been proposed that the on-pathway intermediate oligomers are polymorphic with structures known to be elusive^1^. Consequently, it is important to describe in molecular detail how oligomers can be converted to fibrillar forms, especially in terms of a given oligomer size, because the size of the initial aggregate is dictated by experimental conditions.

Although our computational ability to describe Aβ oligomers and fibrils have improved dramatically^21^,^22^, the description of Aβ fibrillization based on all-atom models has been extremely challenging owing to our inability to capture the multi-scale nature of the force-field, the very long time scales of fibrillization (much longer than for protein folding) and, most importantly, the variety of polymorphic structures^23^. For Aβ_17-42_ peptides, all-atom simulations examining the stability of preformed stacked structures have been performed^24^,^25^, and fibril elongation by monomer addition to a preformed fibrillar structure has been simulated for an extended period of time^26^, which showed that monomer addition and reorganization near a preformed structure containing as few as one monomer requires extensive simulation time. The coarse-grained simulations using OPEP force field for Aβ_17-42_ trimers proposed the 14 dominant oligomer clusters and one less-frequent fibril-like state for further studying binding affinity with five drugs by atomistic simulations^27^. In our previous study^28^, we described the entire fibrillization process for 8 Aβ_17-42_ peptides starting from randomly denatured structures, progressing through the formation of oligomeric intermediates and leading to the formation of the U-shape fibril structures, which were consistent with the progression observed in other experiments^13^,^29^.

Here, we suggest that structural insight into how oligomers can convert into polymorphic fibrillar forms depends on the given number of chains in a system, based on a coarse-grained model of Aβ_17-42_ peptides. This work presents direct observations of transient on-pathway structural intermediates and also provides valuable insight into the generation of stable off-pathway oligomers caused by the polymorphism inherent to fibrillar structures from assembled Aβ_17-42_ peptides, depending on size.

## Results

### On-pathway fibrillization was enhanced by slight modification of PRIME20 geometry

We performed discontinuous molecular dynamics (DMD) simulations using the coarse-grained PRIME20 model for spontaneous fibril formation of Aβ_17-42_ peptides in a box (1 mM concentration) starting from a random configuration (see Methods). In our previous work simulating Aβ_17-42_ peptides and showing multiple kinetic pathways, we used an augmented PRIME20 by incorporating parallel preference constraints and an enhanced salt-bridge interaction into the original PRIME20^28^. These modifications to PRIME20 significantly reduced the complexity associated with sampling the energy landscape to enhance on-pathway fibrillization. Here, we added another modification to the model-geometry of the twenty amino acids by slightly adjusting the pseudo-bond lengths and squeeze distances between side-chain centroids and united backbone atoms (see Methods). This modification to the model-geometry made the side-chains more flexible, making the conversion process and escape from meta-stable states easier. These additions were a significant improvement for the structural conversion toward homogeneous fibril formation and allowed for diverse structures including highly disordered oligomers at an initial collapsed stage.

### Polymorphism of fibrillar structures for a system with 8 Aβ_17-42_ peptides

Before investigating the consequence of oligomer size, we examined the polymorphism of fibrillar oligomers with an eight peptide system; the number of chains (NC) was 8 in an 1 mM box (L^3^ = (237 Å)^3^). We simulated at two constant reduced temperatures T* (k_B_T/ε_HB_) = 0.198 and 0.20, which are both slightly below the “fibrillization temperature (T_f_ ~ 0.205 for NC = 8),” the temperature above which fibrils cease to form spontaneously. Additionally, we employed a new technique, which uses two alternating temperatures (T_1_, T_2_) during a simulation to produce well-ordered fibrillar structures (see Methods). We adopted four pairs of alternating temperatures (T_1_, T_2_) = (0.204, 0.198), (0.206, 0.20), (0.208, 0.20) and (0.21, 0.20). Finally, we performed 50 independent runs at each constant temperature or alternating temperature pair such that a total of 300 trajectories were run until 700 billion collisions was reached (t/σ(k_B_T/m)^1/2^ = t* ~ 94,000 for NC = 8).

Of the various structures obtained from the 300 independent trajectories, we needed to classify the diverse structures: fibrillar, β-helix, disordered or hybrid structures. A hybrid structure was defined as an aggregate containing of two different structures or heterogeneously aligned with similar structures. Additionally, fibrillar structures were also classified based on their diverse polymorphic states. Classification by eye from structural snapshots is unsatisfactory because many structures exhibit partial order, hybrid structures or out-of-register β-sheets. We devised a measure to classify fibrillar structures and check similarity with known fibril structures in deposited PDB structures. Measuring the similarity between a known PDB structure and a structure from our simulation was estimated with the Pearson correlation coefficient (*r*) (see Methods).

**Figure 1:**
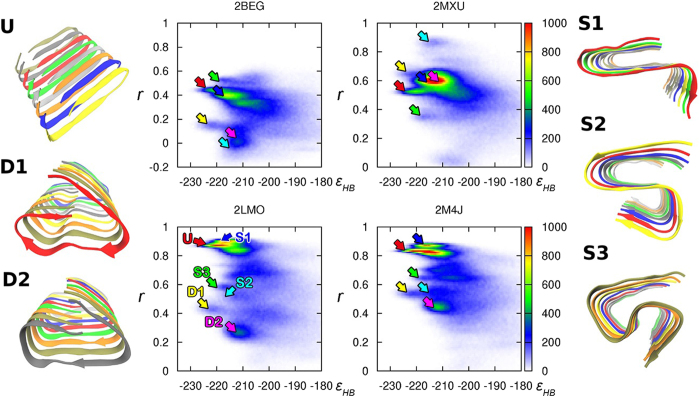
The population heat maps for the similarity measure (the Pearson correlation coefficient *r*) with 2BEG, 2MXU, 2LMO, and 2M4J versus the total interaction energy in units of ε_HB_ (energy strength of a hydrogen bond). Six selected fibrillar structures showing polymorphisms are denoted as U-shape (U), S-shape (S1, S2, S3) and other fibrillar structures (D1, D2). Various colored arrows indicate the position of each structure in the heat map. Each structural figure was obtained from averaging over five consecutive frames during Δt* ~ 10 in simulations using the averaging method in the VMD software.

Six fibrillar structures (Fig. 1) that were highly in-registered and called U-shape (U), S-shape (S1, S2, S3) or other fibrillar structures (D1, D2) were selected for further investigation. The population of those fibrillar structures were evaluated over all six types including slightly out-of-registered structures; U(16.2%), S1(7.2%), S2(3.1%), S3(2.6%), D1(2.6%), D2(7.8%). We estimated the average energies for only highly in-registered structures to check differences among the well-ordered structures; U(−221ε_HB_), S1(−215ε_HB_), S2(−213ε_HB_), S3(−215ε_HB_), D1(−221ε_HB_), D2(−211ε_HB_). Other minor fibrillar structures (Supplementary Fig. 1) were rarely observed, D3(0.6%) and D4(1.3%), and less stable, D1(−206ε_HB_) and D3(−210ε_HB_). Among four D structures, D1 has the lowest total interaction energy and D2 is the majority structure. Hence we disregarded D3 and D4 in further examination. We compared the well-ordered fibrillar structures with known PDB structures: 2BEG (17–42 residues by Lührs *et al*.)^30^ and 2MXU (11–42 residues by Xiao *et al*.)^31^ for the Aβ_42_ peptide and 2LMO (9–40 residues by Petkova *et al*.)^29^, which was selected from a series of PDB structures (2LMP, 2LMQ, 2LMR), and 2M4J (1–40 residues by Lu *et al*.)^32^ for the Aβ_40_ peptide. The population heat maps for the similarity measure (*r*) versus the total interaction energy of a system (Fig. 1) were obtained from frames during the last 30% of the simulation time of 300 trajectories. We observed a highly populated region (red color), *r* > 0.8, on the two heat maps for 2LMO and 2M4J, which notably corresponded to two fibrillar structure regions, the S1- and U-shape structures. The U-shape has a lower total interaction energy than the S1-shape. The 2LMO and 2M4J PDB structures are Aβ_40_ fibrils; therefore, the similarity measures were evaluated over residues 17 to 40. As a result, owing to neglecting the two C-terminal residues I41 and A42, evaluation of the similarity measure for the two structures U and S1 with known PDB structures naturally generated similar values except for slight directional differences for the C-terminal residues. High similarity of the intra-peptide state for a peptide with 2LMO and 2M4J PDB structures indicated that our U-shape structure is similar to the known structures for synthetic (2LMO)^29^ and brain-derived (2M4J)^32^ Aβ_40_ fibrils provided by Tycko and coworkers. Notably, the intra-peptide U-shape structure for synthetic Aβ_40_ fibrils is also similar to that of Aβ_42_ fibrils provided by Smith and coworkers^13^. All of the heat maps revealed that the in register U-shape has the lowest energy, implying that it is the most stable fibrillar structure. Interestingly, the S-shape structure called S2 has high similarity with 2MXU, which is a recently suggested Aβ_42_ fibril structure. Hence, our simulation successfully produced another known fibril PDB structure.

**Figure 2:**
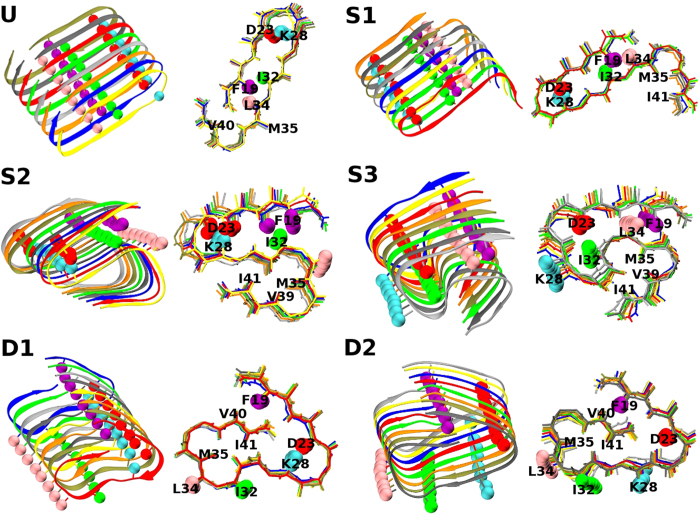
Detailed structures depicting the positions of the side-chains for the six selected fibrillar structures. Each has a cartoon diagram and stick figure with side-chain spheres: F19 (purple), D23 (red), K28 (cyan), I32 (green) and L34 (pink). Other side-chain spheres are not shown for easy viewing. The selected S2 is not fully in-register because we do not have a perfectly in-register S2 structure from our simulations for NC = 8. The other five structures are perfectly in-register. Actual figures were made by averaging five consecutive snapshots using a tool provided by the VMD software.

The detailed structures regarding the side-chain positions for the six structures are depicted in Fig. 2, showing differences in hydrophobic interactions (F19 and I32, L34) and salt-bridges (D23 and K28). The detailed positions of the side-chains in U and S1 are very similar to those of the synthetic Aβ_40_ fibrils (2LMO) provided by the Tycko group. However, the salt-bridge is not formed in S3 and D2, and the hydrophobic interaction pattern in D1 and D2 differ notably from those in U and S1. The S3 and D fibrillar structures do not show high correlations with known PDB structures, despite being highly in register and possessing lower energies than hybrid or disordered structures. It is uncertain whether the S3 and D structures are intrinsic fibrillar structures or kinetically trapped states accessible only in the coarse-grained model. However, it is worth noting that the morphology with the highest stability does not always determine the most frequent fibril structure because ‘fibril polymorphism is under kinetic control’ implying that fibrillation can occur via kinetically more accessible intermediates^33^. Hence we presume that this additional polymorphic feature might result in highly complex, heterogeneous and hybrid fibril morphology, which might be observed in experiments at the initial incubation stage before the seeding and filtration process.

In addition to highly in-registered structures (Fig. 1), we also observed diverse structures with NC = 8, including partially out-of-register U, S2, S3, D1 and D2 structures (Supplementary Fig. 2), disordered, β-helix structures (BH), and hybrid structures (Supplementary Fig. 3). BH and hybrid structures are meta-stable, demonstrating that the conversion toward homogeneous fibrillar structures is an extremely difficult process in our simulations. Consequently, we regard the BH and hybrid structures as types of off-pathway oligomers.

### Improvement in fibrillization by enhanced thermal fluctuation

We checked the influence of temperature on fibril formation at two constant temperatures and four alternating temperature pairs. The heat maps for the similarity measure with five known PDB structures (Supplementary Fig. 4) and the probability histogram for the populations of defined structures (Supplementary Fig. 5) were evaluated at each temperature. Markedly, the fibrillar structures were frequently observed using the alternating temperature method, and the U-shape fibrillar structure was a dominant species. The heat maps and histogram figures show that the alternating temperature technique is an effective method for enhancing on-pathway processes in fibril formation. Having established this methodology, we are now ready to study the oligomerization and diverse polymorphic fibrillar structures that depend on oligomer size during on-pathway fibrillization, for which we adopted the enhanced salt-bridge, parallel preference constraints, a new parameter set for PRIME20 geometry and a new simulation technique for enhancing thermal fluctuations.

### Oligomer and fibrillar structures depend on oligomer size

In addition to the NC = 8 system, we performed further simulations at six temperature ranges with different numbers of Aβ_17-42_ peptide chains at the same concentration (1 mM): NC = 1, 2, 3, 4, 5, 6, 7, 10 or 12 in boxes (L^3^ = (118 Å)^3^, (149 Å)^3^, (171 Å)^3^, (188 Å)^3^, (202 Å)^3^, (215 Å)^3^, (226 Å)^3^, (255 Å)^3^ or (271 Å)^3^). By performing 10 runs at each temperature, we completed a total of 60 independent runs for each NC. For NC = 1, only a disordered monomer is observed at this simulation temperature condition, which corresponds to the unstructured state of the Aβ peptide at human body temperature. For NC = 2, two separate disordered monomers were observed in most simulations, and weakly coupled dimers were rarely observed, indicating that the observed dimers were still unstable under this simulation condition. For the rough estimation of the folding temperature for monomers and the dimerization temperature for dimers, we performed additional simulations at low temperatures; T* = 0.16, 0.17, 0.18, 0.19, 0.195 for NC = 1 and 0.18, 0.19, 0.195 for NC = 2. The population heat maps and representative structures (Supplementary Fig. 6) display different patterns depending on temperature for monomers and dimers. Monomers form β-hairpins at significant low temperatures T* = 0.16 & 0.17 and dimers form β-helical structures at T* = 0.18 & 0.19. From the heat maps and structures, the folding or fibrillization temperatures are roughly estimated as T_f_ ~ 0.18 for NC = 1 and T_f_ ~ 0.195 for NC = 2, which are below the six temperature ranges used in our main study for larger oligomers.

**Figure 3:**
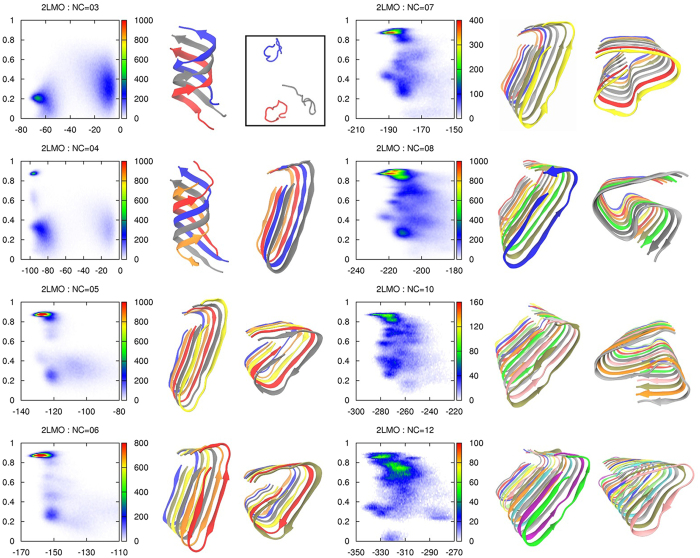
The population heat maps for the similarity measure with 2LMO versus the total interaction energy of a system and two representative structures for NC = 3, 4, 5, 6, 7, 8, 9, 10 and 12. The left figure is the dominant structure, and the right figure is the next-populated or lower energy structure.

**Figure 4:**
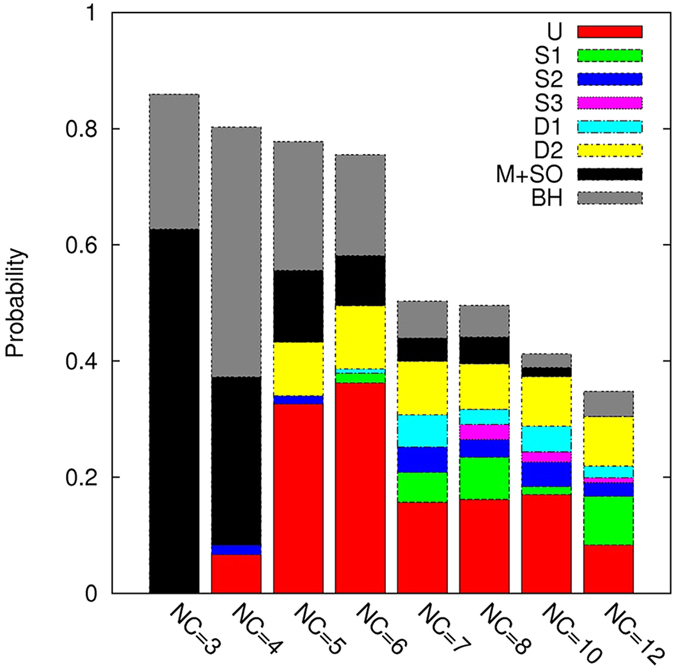
Histogram plot for probabilities of the diverse structures with different numbers of chains from NC = 3 to NC = 12. Six fibrillar structures, U-shape, S-shape (S1, S2, S3) and the other diverse structures (D1, D2), are presented. Fractions of monomers and smaller oligomers (M + SO) and β-helix aggregates (BH) are given in the histogram to show the structural differences between the oligomers depending on size.

In Fig. 3, we present the population heat maps for similarity with 2LMO and two representative structures (left figure is the dominant structure and right figure is selected from either the next populated or lower energy structure) for each NC = 3, 4, 5, 6, 7, 8, 10 and 12. An additional five various structures are shown for each NC (Supplementary Figs 7 and 8). The populations of diverse structures at each NC were evaluated, and the probability histogram plot is presented in Fig. 4.

The heat map for NC = 3 depicts two divided regions. The widely distributed region at higher energy corresponds to separate disordered monomers (63%), and the structure at the lower energy region is a β-helix (BH) (23%). This trimer BH is actually a globally helix-like β-sheet maximizing inter-chain hydrogen bonds. For small oligomers such as NC = 2 or 3, we observed random monomers at a high probability. Hence, oligomerization toward nucleus formation is still an uphill process in a free energy landscape under the present high temperature condition.

For NC = 4, the most frequent structure was also the BH. Although the left figure for NC = 4 in Fig. 3 is a tetramer BH, the most common BH structure for NC = 4 was an aggregate of a trimer BH and a monomer. In Supplementary Figs 7 and 8, we attempted to present diverse BH structures including a monomer, dimer, trimer, and tetramer and their aggregates. A small U-shape region at *r* = 0.87 also appeared on the heat map, although U and BH structures have similar energy scales in our simulation under the PRIME20 force field. Strictly speaking, the U-shape shows slightly lower energy, but BH (43%) is far more populated than the U-shape (7%) as shown in Fig. 4, which means that BH is a kinetically favored structure. We did not observe any conversion from a trimer BH to a U-shape. Consequently, the trimer BH structure, which is kinetically favored and is the primarily observed species in our simulations, can be regarded as a potent off-pathway oligomer.

For NC = 5 and 6, we observed that the dominant fibrillar structure was a U-shape (32% for NC = 5 and 36% for NC = 6) at near *r* = 0.87, and the minor structure D2 (9% for NC = 5 and 11% for NC = 6) was observed at near *r* = 0.25. The selected U and D2 structures for both NC = 5 and 6 are shown in Fig. 3. The U-shape structures, which have the lowest energy, can be formed easily with significantly less trapping into other fibrillar structures. Among the 45 total U-shape fibrillar structures for both NC = 5 and 6, we observed that 34 U-shape structures were converted from partial or full S-shape structures, which means that a small S-shape oligomer is not stable alone. These multiple pathways for structural conversion mechanisms are consistent with our previous study^28^. Because oligomers with NC = 5 and 6 can be easily converted into U-shape fibrillar structures in our simulations, we suggest that a pentamer or a hexamer called a paranucleus^34^,^35^ might be a potential nucleus size or an on-pathway intermediate for easy structural conversion and further fibril growth. For the special role of NC = 6 oligomers, there were some investigations like a simple mathematical model for prion replication by NP process^36^ and thermodynamic stability studies of alanine rich β-sheet oligomers^37^ and Aβ-tau complexes^38^, which supported that a hexamers is the minimal oligomeric size in forming fibril-like structures. Here our study directly revealed the conversion process into fibrillar structure having a minimal nucleus as a paranucleus.

We checked the time evolution of the secondary structures (α-helix, β-strand, coil or turn) for NC = 2, 3, 4 and 6. In Supplementary Fig. 9, the average β-strand content over 60 independent runs shows almost saturated values after 700 billion collisions. The β-strand content for NC = 6 is 57%, which is close to the final value 52% of Aβ_40_ and Aβ_42_ fibrillation^39^. We observed no α-helix content at early or intermediate stage contrary to their experiments owing to our parallel preference constraints and high temperature condition. For the dimer, we observed very high probability of coil or turns (95%) and very low β-strand content (4%), which implies more unstructured comparing to simulations of Aβ_42_ dimer having 80% coil/turn and 11% β-strand^40^.

For NC = 7, the U-shape (16%) is still the dominant structure, but various fibrillar structures such as S1 (5%), S2 (4%), D1 (6%), and D2 (9%) were more frequently observed (Fig. 4 and Supplementary Fig. 8). The right representative figure for NC = 7 (Fig. 3) was selected from a D1 structure near *r* = 0.43 because it exhibited very low energy comparable to the U-shape. For NC = 8, the U-shape (16%) was dominant, and S3 (3%) near *r* = 0.6 was selected as the representative figure. For NC = 10, the U-shape (17%) was still the most common. However, for NC = 12, the probability of the U-shape (8%) was decreased and comparable to the S1-shape (8%). We did not perform simulations for NC = 9 or NC = 11 because it would take too much time (2 months in real time on one cpu core for a run with NC = 8) and need extensive computing ability to run all systems with different number of chains. However the heat maps in Fig. 3 and the histogram probability in Fig. 4 did not display any significant changes among NC = 8, 10 and 12.

Although diverse fibrillar structures were observed in our simulation, fewer well-ordered structures were obtained for larger chain numbers of NC = 10 or 12 (Supplementary Fig. 8) because hybrid or disordered structures were more frequent, implying that conformational conversion toward a homogenous U-shape fibril structure from disordered oligomers is a difficult process for large oligomers. Consequently, we suggest that the initial aggregated size of disordered oligomers by hydrophobic collapse might be one of the important factors triggering the on- or off-pathways, which depend on the experimental environment including the concentration, temperature and buffer. The direct formation of a meta-stable paranucleus without an intermediate formation step of a meta-stable trimer will enhance on-pathway fibrillization.

### Off-pathway oligomers

In this work, we applied biased constraints, a new set of parameters and a new simulation technique to enhance on-pathway fibrillization. Nevertheless, we still observed diverse oligomers that were not converted into fibrillar structures. Those oligomers include disordered and partially ordered structures, BH structures, and hybrid oligomers. Although the former two types might be converted to the ordered structure after a longer simulation time, the complex aggregates of BH and hybrid heterogeneous structures persist as off-pathway oligomers.

Our simulations carefully suggest that the BH structure, primarily as a trimer, appears to be a meta-stable off-pathway oligomer and plays an important role in oligomerization for both low-molecular weight (LMW) oligomers and high-molecular weight (HMW) non-fibrillar oligomers. We presume that meta-stable BH structures might be a building block for the formation of larger off-pathway non-fibrillar oligomers containing trimers (Supplementary Figs 7 and 8). This is reminiscent of the oligomerization process using trimer building blocks, which leads to off-pathway toxic species^2^,^41^.

We did not frequently observe intra-peptide hydrogen bonds leading to a β-hairpin structure, which can also generate off-pathway oligomers such as the soluble oligomer with β-hairpins^10^, the cylindrin trimer^42^, the β-hairpin trimer^12^ and the hexameric peptide barrel^14^. Inter-hydrogen bonds were clearly preferred in our simulations, which is reasonable as the simulation maximizes both hydrogen bonds and the steric zipper interface by hydrophobic and van der Waals interactions. Nevertheless, we assume that one reason for the substantial preference for inter-peptide hydrogen bonds can be attributed to the parallel preference constraints enhancing fibril formation. In other words, our simulations might not significantly access the structural region of anti-parallel β-hairpins. For this reason, we obtained the BH structure, which is different from other suggested trimers with β-hairpins^12^,^42^.

The hybrid fibrillar structures, which rarely or never converted to the homogeneous structure, might be prevalent in HMW fibrillar oligomers. We expect this hybrid structural feature might persist for an extended period of time. If this type of hybrid structure grows to a fibril, we expect to observe a highly curvilinear or worm-like chain fibril morphology, which might be easily fragmented by external fluctuations such as sonication and agitation, based on the results of Iowa mutant fibrils^43^. Otherwise, the oligomer might require substantial additional time to convert into the homogenous structure. Because it is unclear if these hybrid oligomers can be converted into homogeneous structures by the fragmentation and growth process, we currently consider these to be off-pathway oligomers. In conclusion, we have two types of off-pathway oligomers: the aggregates of meta-stable BH and the hybrid fibrillar oligomers, with more exposed hydrophobic residues. We presume that these two types of off-pathway oligomers might have different synaptotoxic impacts when interacting with membranes or synaptic receptors^3^.

### All-atom explicit MD simulations for trimer, tetramer and pentamer structures

We checked the structural validation for dependence on oligomer size by evaluating the stabilities via all-atom simulations for trimer BH, tetramer BH, trimer U, tetramer U and pentamer U structures. Positions of all-atoms were generated by applying the MODELLER program^44^ to our coarse-grained structures obtained from simulations. Molecular dynamics simulations were performed with AMBER/ff99SB force field^45^ and explicit TIP3P water for 200 ns at three temperatures 278 K, 298 K and 310 K. (See Method) Observations related to stability, such as RMSD, RMSF (backbone atomic positional fluctuations) and binding energy (intermolecular energy), were evaluated (Supplementary Figs 10 and 11). From the all-atom simulations, we observed that trimer BH was more stable than trimer or tetramer U, which strongly supports our results of PRIME20 simulations.

### NP and NCC depending on thermal fluctuation

**Figure 5:**
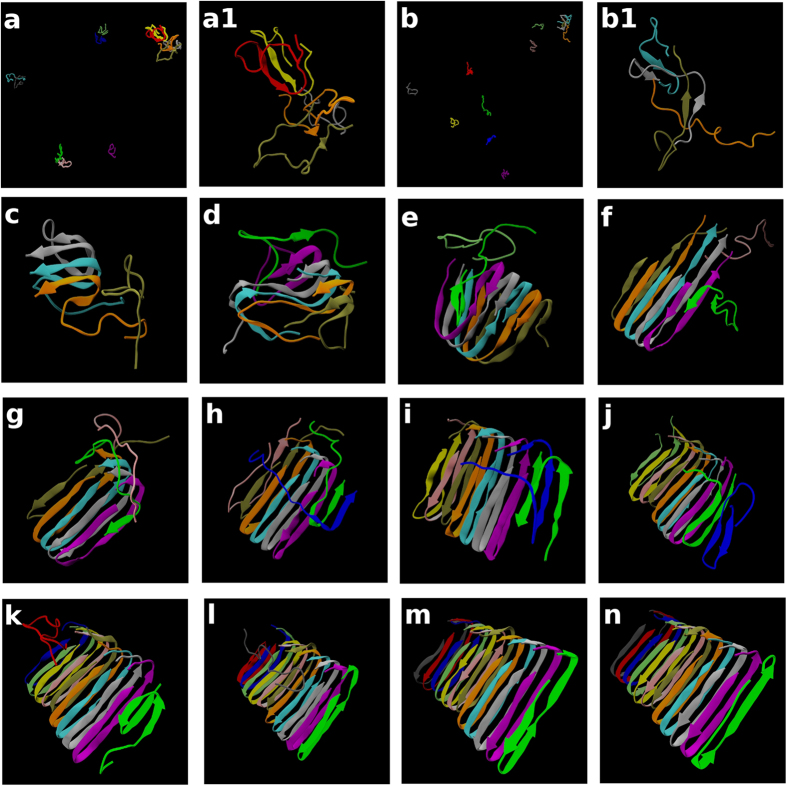
Snapshots for a trajectory-forming U-shape structure for NC = 12. Snapshots are taken at (**a**) t* = t/σ(k_BT_/m)^1/2^ = 2828, (**b**) 11005, (**c**) 11040, (**d**) 11280, (**e**) 11430, (**f**) 11649, (**g**) 11692, (**h**) 11975, (**i**) 12777, (**j**) 13645, (**k**) 16652, (**l**) 17729, (**m**) 18632, and (**n**) 54494 (700 billion collisions). Although (**a**) and (**b**) display whole systems of 12 peptides whose larger oligomers are zoomed in (a1) and (b1), snapshots from (**c**) to (**k**) show only large oligomer parts without separated monomers for easy viewing. Snapshots from (**l**) to (**n**) display one oligomer with all 12 chains.

In our previous work, we presented two pathways for the conversion from disordered oligomers to a U-shape fibrillar structure, in which both conversions are essentially performed under NCC^28^. Although NCC was still dominant in all of our simulations, a partial NP process was observed in conditions of high thermal fluctuation in which free monomers were available for a long time. We present two trajectories with 12 snapshots for NC = 12 and 10 (Fig. 5 and Supplementary Fig. 12). We observed monomer additions to a template and conversion into a U-shape; for example, the red chain (Fig. 5k,l) and gray chain (Fig. 5l,m) for NC = 12 exactly represent the dock-and-lock mechanism^26^,^46^, and this dock-and-lock to a template is a main feature of the NP mechanism. Interestingly, both two snapshot-sequences displayed the structural conversion from the S-shape to the U-shape (Fig. 5e–g and Supplementary Fig. 12g–i). Notably, the number of S-shape chains in each trajectory is 5 (Fig. 5) and 6 (Supplementary Fig. 12), which, again, supports the hypothesis that the conformational conversion occurs easily for a pentamer and a hexamer. It is worth noting that we observed very nice homogenous in register fibrillar structures for larger NC under high thermal fluctuation, which implies that the NP process plays an important role in the formation of straight fibril morphology.

### Quaternary structure

To our surprise, we observed stacked U-shape structures for NC = 12. We performed more simulations to 1 trillion collisions for systems with NC = 12. Among 60 independent runs, we obtained two trajectories showing stacked U-shape structures that were similar to the two-fold symmetry structure (2LMO) provided by the Tycko group^29^. However, the two independent trajectories clearly show different pathways. The stacked U-shape was formed via conformational changes in two parts within a collapsed disordered oligomer (Supplementary Fig. 13). Two separately ordered oligomers were merged and adjusted to form a stable stacked structure (Supplementary Fig. 14).

## Discussion

The powerful combination of a coarse-grained (four-sphere-per-residue) protein model, PRIME20, and discontinuous molecular dynamics significantly facilitated tracking the aggregation process of peptide chains (Aβ_17-42_). Aβ_17-42_ is a good stand-in for its longer parent protein, Aβ_42_, because it contains the two hydrophobic stretches that drive the aggregation of Aβ_42_ as well as the turn region. In fact, we observe similar structural features, such as the meta-stable BH trimer for NC = 3 and U-shape structures for NC = 6, in preliminary Aβ_42_ simulations with NC = 3, 4 and 6, which should be performed for a much longer simulation time using more independent runs owing to the enormously increased complexity of the energy landscape.

Owing to the relatively high concentrations (1 mM), we could not follow the real fibrillization mechanism including lag time, nucleus formation, conformational conversion and the elongation process. Instead, we present the successful simulations that formed fibrillar structures from a given number of chains and analyzed oligomerization and fibrillization depending on oligomer size. It is practically impossible in simulations to control the physiological conditions corresponding to the generation of different stable building blocks such as dimers, trimers or paranuclei. Those building blocks are suggested to play an important role in the formation of larger toxic off-pathway oligomers or on-pathway fibrillization. Nevertheless, without any adjustment of the simulation conditions, our work provides meaningful insight for two characteristic oligomer sizes, trimers and paranuclei. Trimers form a meta-stable β-helix structure that can be used as a building block for larger off-pathway oligomers. Pentamers or hexamers (paranuclei) are potent on-pathway intermediates that are converted into a fibrillar nucleus for further fibril growth.

In our extensive simulations, we implemented two biased constraints, a new parameter set and a new temperature technique for enhancing on-pathway fibrillization, which implies that many possible transient states might be missed. Those missed transient structures may have α-helices and intramolecular β-hairpins, which were proposed in the extensive atomistic simulations for an Aβ_40_ dimer^47^. Nevertheless, we show a variety of polymorphisms in structure depending on size. If we turn off the parallel preference constraints, we would generate far richer oligomer structures owing to the intra-peptide hydrogen bonds and antiparallel β-sheets, which are suggested to be important for off-pathway oligomer formation^12^,^16^,^42^. In our preliminary simulations without the parallel preference constraints, we observed highly complex oligomers with high portion of intramolecular β-hairpins at wide temperature ranges and even α-helices at low temperature. To clarify structural features and conversion mechanism of these complex oligomers, far more extensive study than this work would be needed. If we turn off the enhanced salt-bridge, the fibrillization process will be significantly retarded because the formation of the salt-bridge is one of the rate-limiting steps during fibrillization^48^. In summary, we performed very extensive simulations but still explored a biased landscape for oligomerization and fibrillization. However, this work is an important step in the exploration of the complex and polymorphic nature of oligomer and fibril structures.

## Methods

### Application of PRIME20

We used discontinuous molecular dynamics (DMD)^49^,^50^,^51^,^52^,^53^,^54^,^55^ by employing the intermediate-resolution (coarse-grained) force field PRIME20^56^,^57^ to investigate the effect of size on the aggregation of the Aβ_17-42_ peptide. PRIME20 is an extension of PRIME (Protein Intermediate-Resolution Model)^54^,^55^ and is designed to be applicable to all twenty amino acid residues^56^. The adequacy and efficiency of PRIME20 has been proven in short peptide systems^57^,^58^,^59^,^60^. A longer peptide system with 26 residues per chain (Aβ_17-42_) has also been successfully described based on a double-well potential, two biases, parallel preference constraints and an enhanced D23-K28 salt-bridge interaction^28^.

### Adjustment of PRIME20 geometric parameters

Each amino acid has a different set of geometric parameters including hard-sphere diameters and pair-interaction ranges, which were reported in our previous paper. The distances from the side-chain spheres to the C_α_, NH, and CO united atoms are carefully designed to ensure that all amino acids remain in an L-isomer form during DMD simulations. PRIME20 geometry parameters were developed in 2010 and 2011 and use the distances between the side chain centroids and C_α_, NH, and CO united spheres as well as various squeeze distances between hard spheres^56^,^57^. Although PRIME20 parameters generate excellent fibrillization processes and final fibril structures^28^,^57^, we observed a slightly higher propensity to form β-sheets even in the early stages of simulation. Therefore, we slightly adjusted the PRIME20 geometry parameters by performing simulations of Poly-X peptides with various trial distance sets. Those new PRIME20 geometry parameters provided maximally allowed regions in Ramachandran plots for small amino acids but still did not allow D-form amino acids. For large amino acids, such as HIS, TRP, TYR, and PHE, the new geometry parameters were determined by considering the consistency with small amino acids in the deviations in the average values for geometry distances over 711 real PDB structures. Hence, those new geometry parameters provided more flexible dynamics for the side-chain spheres. As a result, in simulations for Aβ_16-22_, Aβ_17-42_, Aβ_40_ and Aβ_42_, we observed less formation of β-sheets at early oligomeric stages of the simulations, and the ordering process became somewhat slow. However, the final well-ordered structures were similar to results from the previous PRIME20 geometry parameters.

We revised PRIME20 geometry (Supplementary Tables 1 and 2), having reported the old version in the previous study. The three geometry distances for 20 amino acids are given in Supplementary Table 1, which are the most critical factor in PRIME20, and the minimum non-bonded distances between a side-chain sphere and other neighboring united backbone spheres called the squeeze parameters are given in Supplementary Table 2.

### Enhancing fluctuations by using two alternating temperatures

We developed a new method using two alternating temperatures. This method first simulates at T_1_ during 5 billion collisions and next at T_2_ during 5 billion collisions. This cycle, with 10 billion collisions, is repeated over an entire simulation. In practice, we took T_1_ above T_f_ and T_2_ below T_f_ to enhance the thermal fluctuations for escaping the highly rugged metastable states of oligomers. The simulations confirmed that in-register homogeneous fibrillar structures are generated much more frequently.

This alternating temperature technique is somewhat similar to the simulated tempering algorithm. However, the original simulated tempering method is not applicable to the present DMD system because thermal fluctuation at each given temperature under the present thermostat is large, resulting in a very high acceptance rate by the Metropolis algorithm. Hence changing the temperature with simulated tempering is almost a random process. That is why we devised a new technique in which a sequential temperature change is applied after a fixed period (5 billion collisions).

### Similarity measure using the Pearson correlation coefficient (*r*)

When we measured the similarity between a known PDB structure and a simulated structure, we constructed two datasets comparing the structures. Datasets of the intra-peptide distances among C_α_ atoms were built for both the PDB and the simulated structure. These intra-peptide distances were evaluated over C_α_ atoms from L17 to V40 for 2LMO and 2M4J (or L17 to A42 for 2BEG or 2MXU), with a pair of C_α_ atoms separated by more than 4 residues along the sequence. Distance datasets for each pair were averaged over all of the chains in each instance for the PDB structure and the simulated structures. Hence, each dataset contains a set of long-range distance values for pairwise C_α_-atoms on a single peptide. Two distance variances were evaluated separately for the PDB and the simulated structure. One covariance between the PDB and the simulated structure was obtained. Consequently, with one covariance and two variances, we can estimate the Pearson correlation coefficient (*r*), which is a similarity measure between the known PDB and the simulated structure. Notably, this new measure does not compare the entire fibrillar structure but averages the intra-peptide state over all peptides in an oligomer structure.

### All-atom explicit molecular dynamics (MD) simulations

We used the pmemd.cuda of AMBER14 MD simulation package with ff99SB force field. The starting system was explicitly solvated with TIP3P water molecules in the rectangular box where the distance to the edge of the solvent box from protein was chosen to be 12 Å and periodic boundary condition was applied. Systems were neutralized to add sodium ions. The particle mesh Ewald method was applied for treating long-range electrostatic interactions, and a 9.0 Å force-shifted cutoff was used for short-range non-bonded interactions. The hydrogen atoms were constrained to the equilibrium bond length using the SHAKE algorithm. We performed 10,000 steps of steepest decent minimization followed by 6000 steps of conjugate gradient minimization. The systems were subsequently subject to 10 ps heating process in which the temperature was gradually raised from 0 K to 310 K under the SHAKE algorithm. After the heating step, the production runs were carried out for 200 ns with 2 fs time step and with NPT ensemble. Temperature and pressure were controlled by a Langevin dynamics thermostat with collision frequency 2 ps^−1^ and weak-coupling barostat with coupling constant 2.0 ps. All trajectories were recorded in every 10 ps. MD simulations for each oligomer system were performed to 20 independent trajectories up to 200 ns time scale with different seed. The binding energy was calculated by MM/GBSA method in the time window of 10~200 ns and the RMSF was also calculated in the same time period. The MD trajectories were analysed by CPPTRJ in AmberTools15.

## Additional Information

**How to cite this article**: Cheon, M. *et al*. Polymorphism of fibrillar structures depending on the size of assembled Aβ_17-42_ peptides. *Sci. Rep.* **6**, 38196; doi: 10.1038/srep38196 (2016).

**Publisher's note:** Springer Nature remains neutral with regard to jurisdictional claims in published maps and institutional affiliations.

## Supplementary Material

Supplementary Information
