# Supplementary material for: Polymorphism of fibrillar structures depending on the size of assembled Aβ17-42 peptides
Source: Sci Rep. 2016 Nov 30;6:38196. doi: 10.1038/srep38196 (PMC5128875; doi:10.1038/srep38196)
Supplement: Supplementary Information [file srep38196-s1.pdf]

Supplementary Information

Polymorphism of fibrillar structures depending on the size of assembled Aβ_{17-42} peptides.

Mookyung Cheon[1,2], Mooseok Kang[1] and Iksoo Chang[1,*]

[1]Center for Proteome Biophysics, Department of Brain and Cognitive Sciences, Daegu Gyeongbuk Institute of Science and Technology (DGIST), Daegu 42988, Korea

[2]Department of Neural Development and Disease, Korea Brain Research Institute, Daegu 41068, Korea

[*]Correspondence should be addressed to I.C. (iksoochang@dgist.ac.kr)

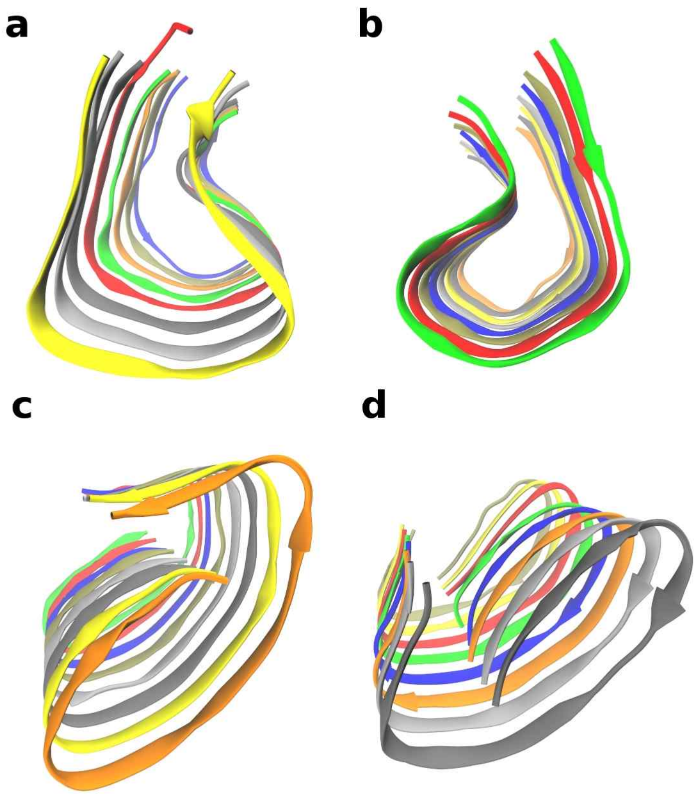

Supplementary Figure 1. The other minor fibrillar structures called D3 (a & b) and D4 (c & d). These are rarely observed and less stable having higher total energy than D1 and D2 fibrillar structures. D3 structure was described as the triangular-shape fibrillar structure in our previous work.[1]

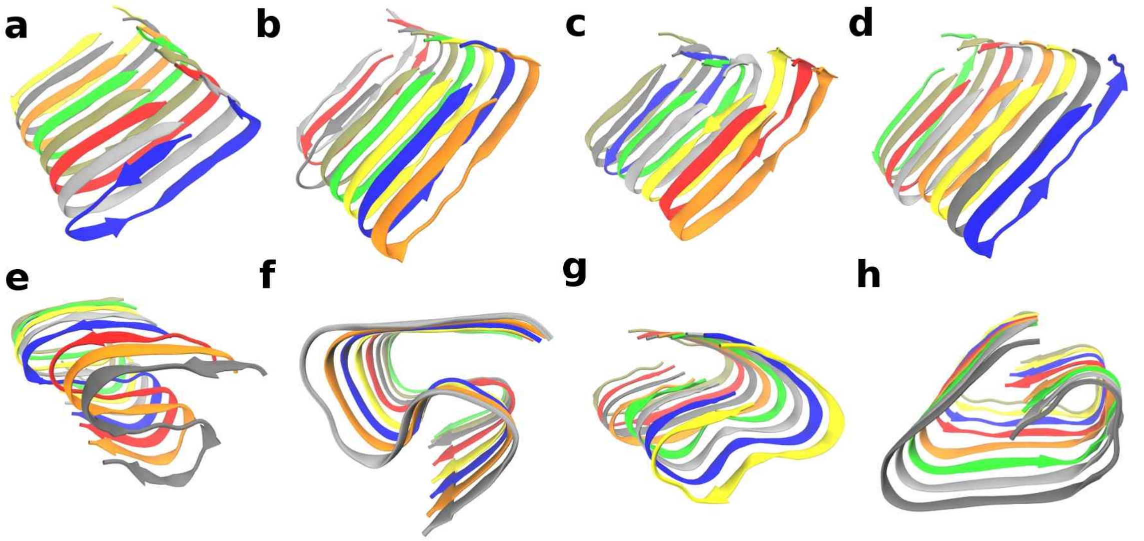

Supplementary Figure 2. Selected snapshots taken at 700 billion collisions of partially out-of-register fibrillar structures in our simulations; (a-d) U, (e) S2 (f) S3, (g) D1, (h) D2 shape.

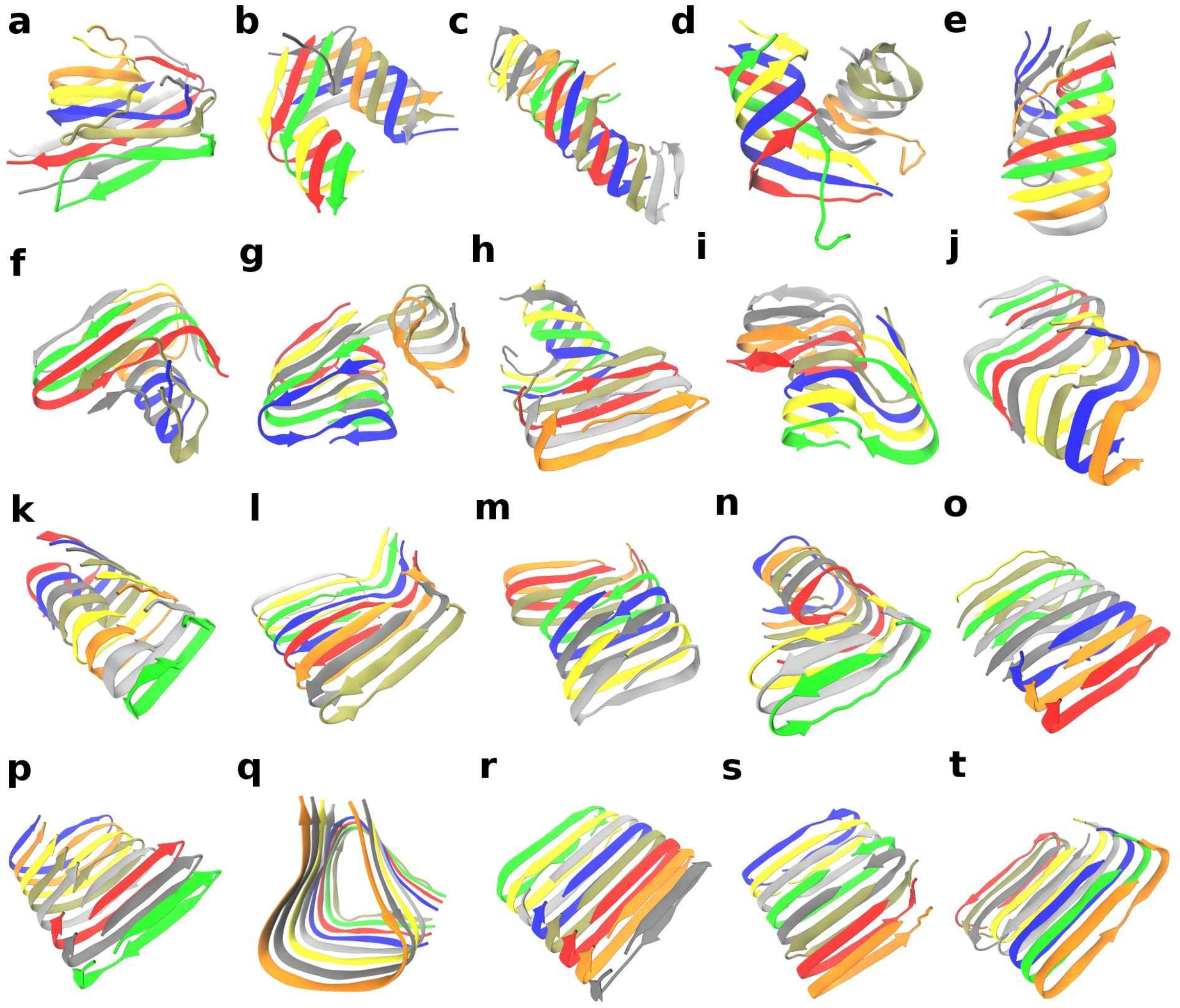

Supplementary Figure 3. Selected disordered (a), β-helix(BH) (b-e), hybrid of β-helix and fibrillar (f-h), hybrid of fibrillar (i-t) structures. The BH structure is different from the generally known β-helix structure observed in the prion fibril structures.[2,3] While the former forms β-sheets in a manner of globally helix-like by maximizing the inter-chain hydrogen bonds, the latter forms a β-helix in a manner of stacking with monomer BH which can be formed by intra-peptide hydrogen bonds.

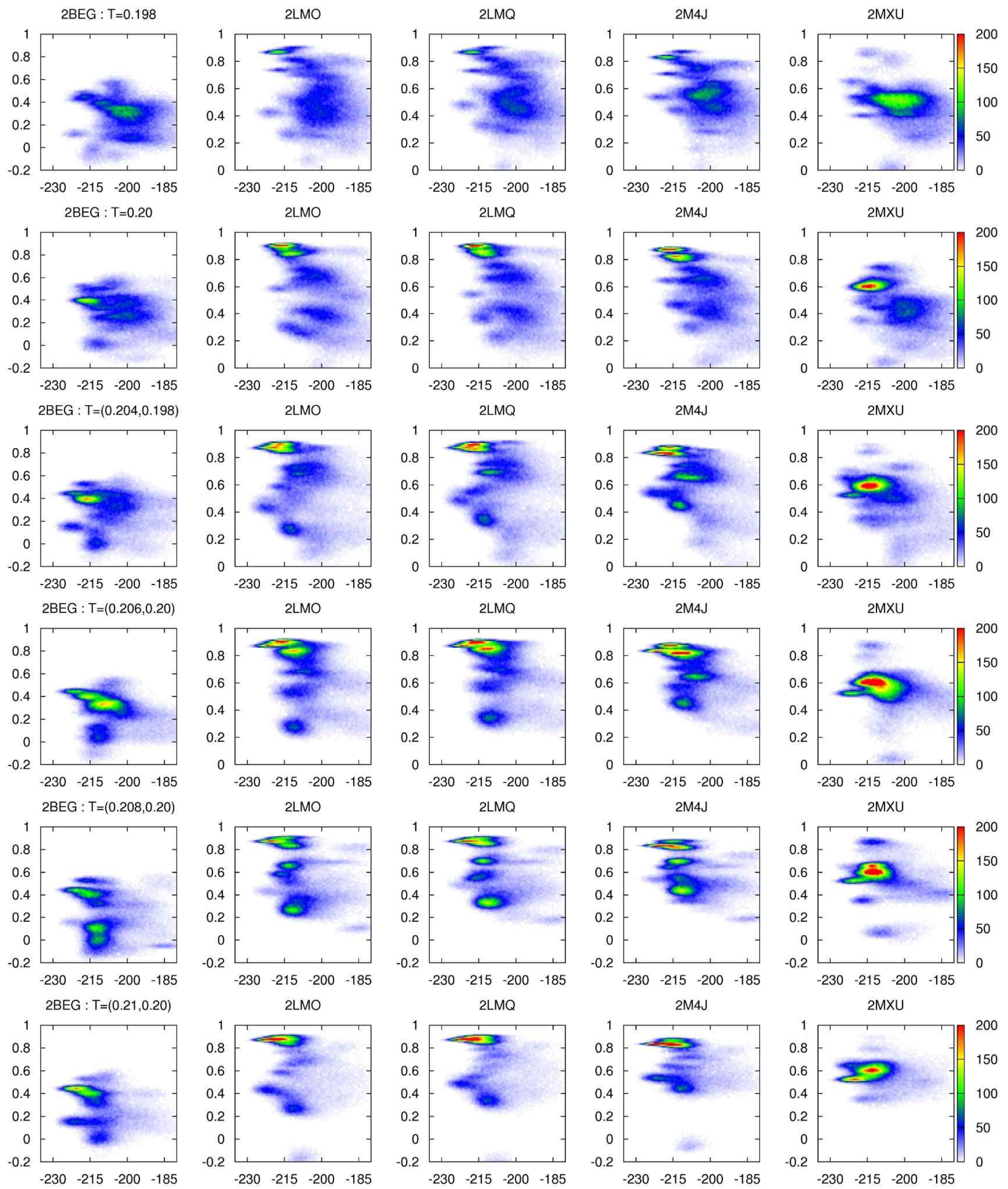

Supplementary Figure 4. The population heat maps of NC=8 systems for similarity measure with five 5 PDBs (2BEG, 2LMO, 2LMQ, 2M4J, 2MXU) at two constant temperatures T*=0.198, 0.20 and four alternating temperature pairs (0.204,0.198), (0.206,0.20), (0.208,0.20), (0.21,0.20). The heat map for 2LMQ PDB with 3-fold symmetry is very similar to that for 2LMO with 2-fold symmetry since we measured similarity for not quaternary structure but average intra-molecular structure for each peptide. Populations of each fibrillar structure are evaluated by counting frames of trajectories in the defined regions on the heat maps corresponding to the six fibrillar structures. In this way, we can exclude counting the hybrid structures (Supplementary Fig.3). Evidently, the regions of the in-registered structures including the U-shape are highly populated when we apply the alternating temperature technique.

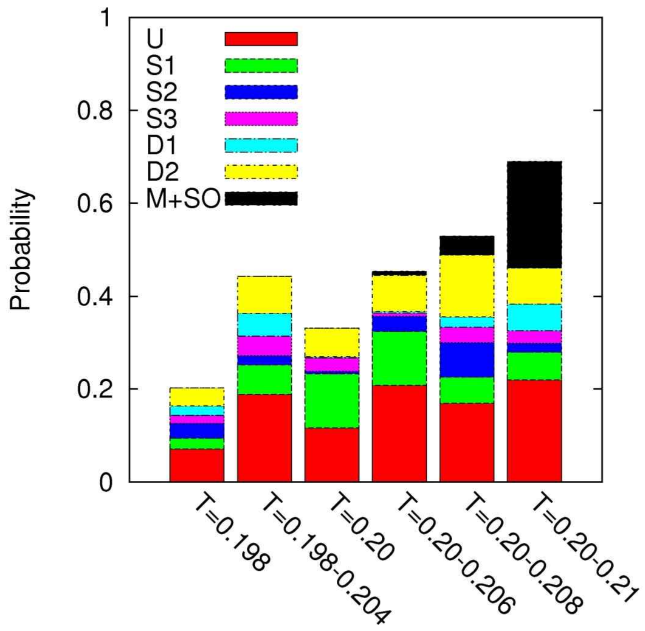

Supplementary Figure 5. Histogram plot for probabilities of diverse fibrillar structures for systems with NC=8, which are evaluated over 50 independent runs for each temperature, at two constant temperature and four alternating temperature pairs. Six fibrillar structures, U-shape, S-shape (S1, S2, S3) and the other diverse structures (D1, D2), are presented and fraction of monomers and smaller oligomers (M+SO) are given in the histogram in order to estimate the influence of large thermal fluctuation. Although simulations at T* (0.21, 0.20) provide the most nice fibril structures, the probability having separate monomers and smaller oligomers (black) becomes increased, which mean that we must not increase T1 too large. Hence simulation with alternatively changing temperature technique indeed reduces the heterogeneity having hybrid or out-of-register structures and notably improves probability of accessing the well-ordered in-registered structures by escaping from traps of meta-stable states.

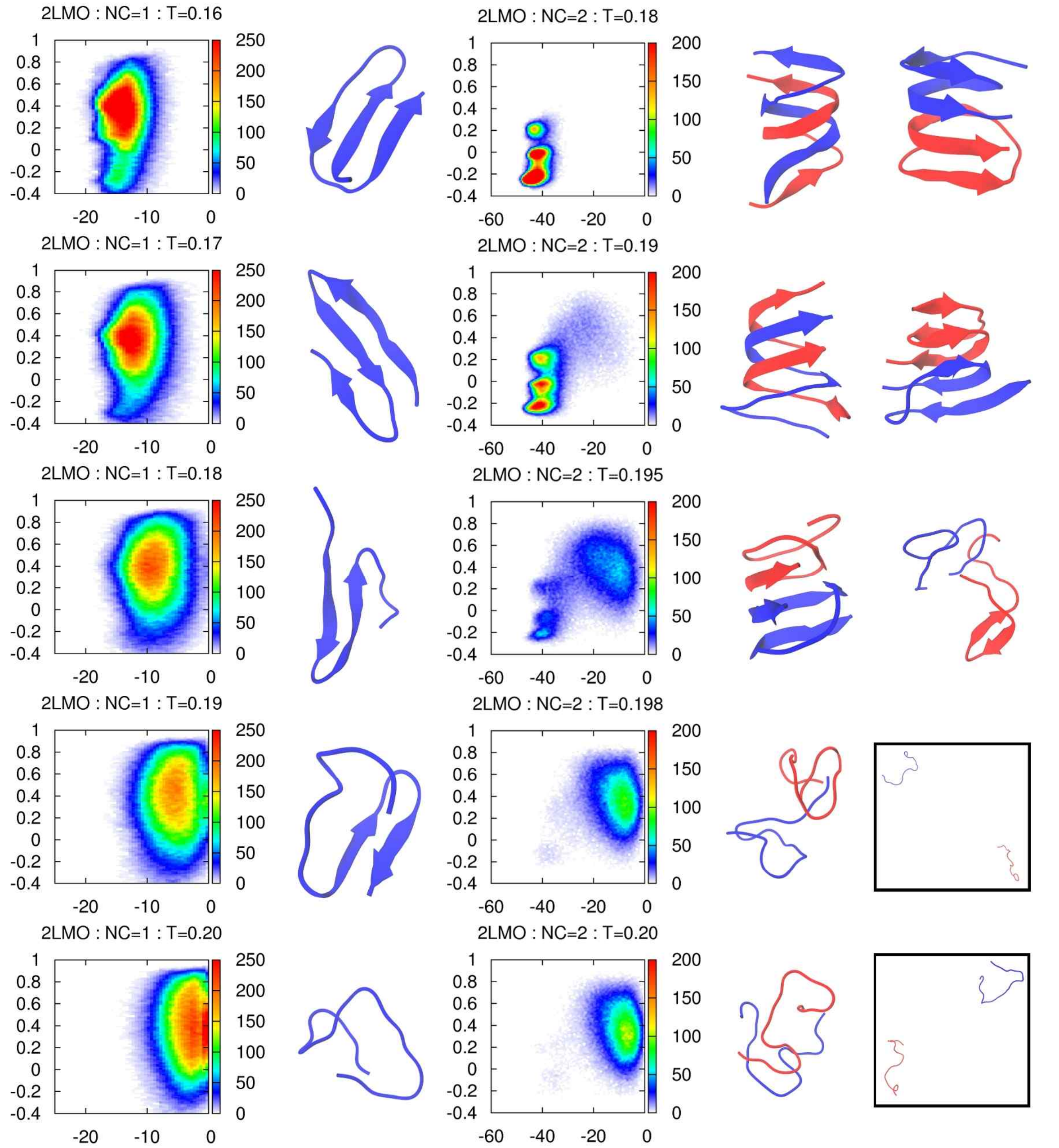

Supplementary Figure 6. The population heat maps for the similarity measure with 2LMO versus the total interaction energy of systems and representative structures at T*=0.16, 0.17, 0.18, 0.19 and 0.20 for NC=1 and at T*=0.18, 0.19, 0.195, 0.198, and 0.20 for NC=2.

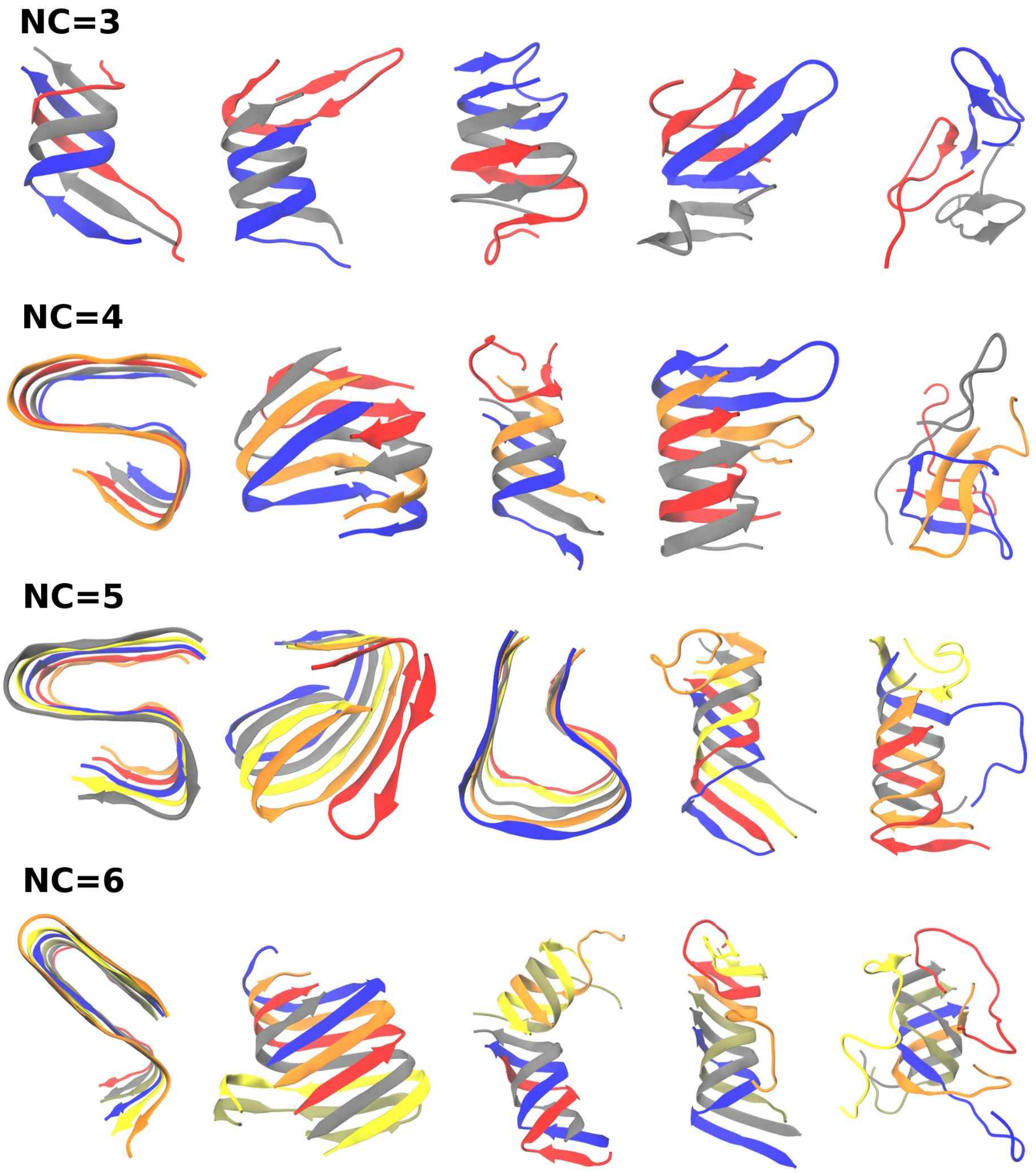

Supplementary Figure 7. Five additional structures are selected to show the diversity of structures including fibrillar, BH and disordered forms for NC=3, 4, 5 and 6. For NC=4, We have only three well-ordered U-shape fibrillar structures among 60 independent runs. Two U-shape structures are directly converted from disordered oligomers by starting in-registering from C-terminal residues. The other one is converted from an aggregate composed of a dimer BH and disordered chains. We observe 26 trajectories having BH structures among 60 runs, 5 tetramer BH and 21 trimer BH + monomer, but there is no conversion from tetramer or trimer BH to U-shape. For BH structures of NC=5 & 6, we observed more complex pentamers or hexamers (22% for NC=5 and 17% for NC=6) composed of various BHs and disordered chains

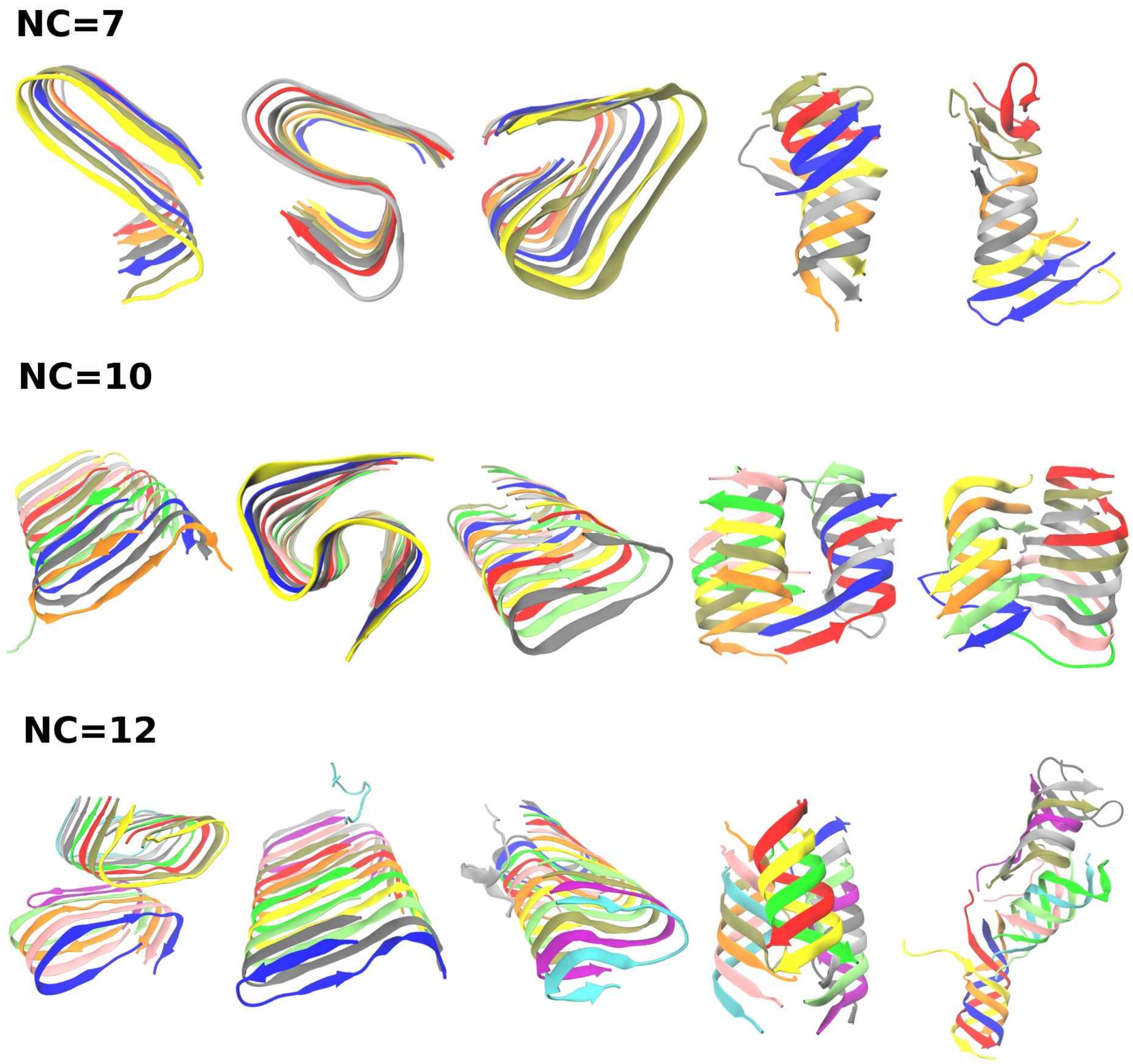

Supplementary Figure 8. Five additional structures are selected including three fibrillar structures and two BH aggregates composed of smaller BHs for NC= 7, 10 & 12.

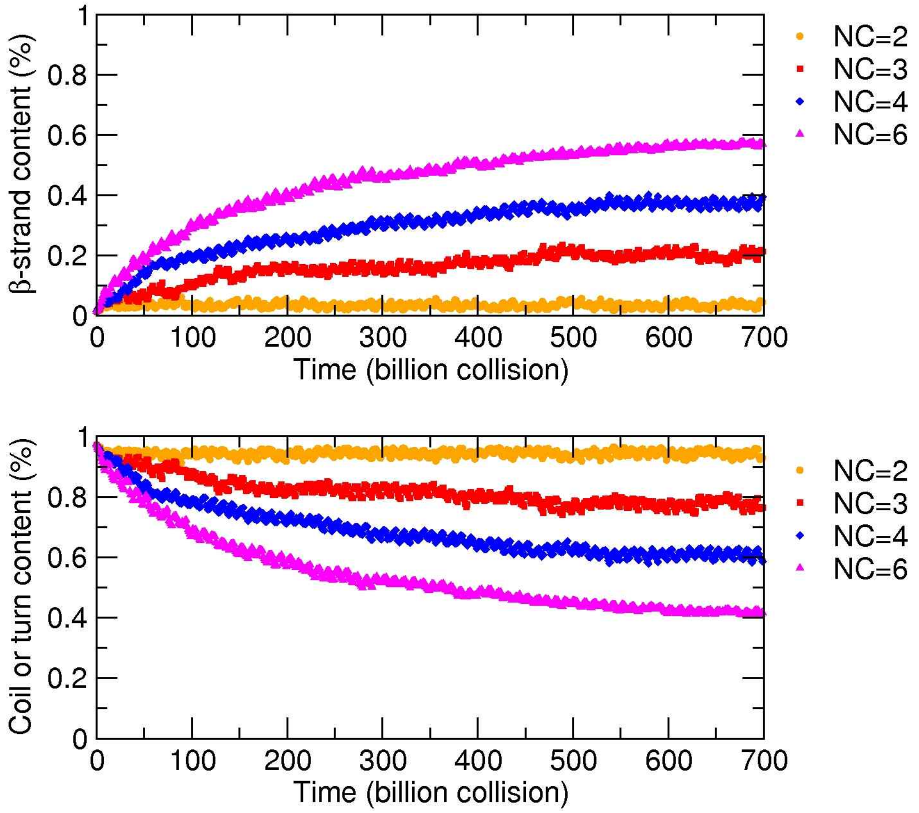

Supplementary Figure 9. The time evolution of the secondary structures (β-strand, coil or turn), which are averaged over 60 independent runs, for NC=2,3,4, and 6.

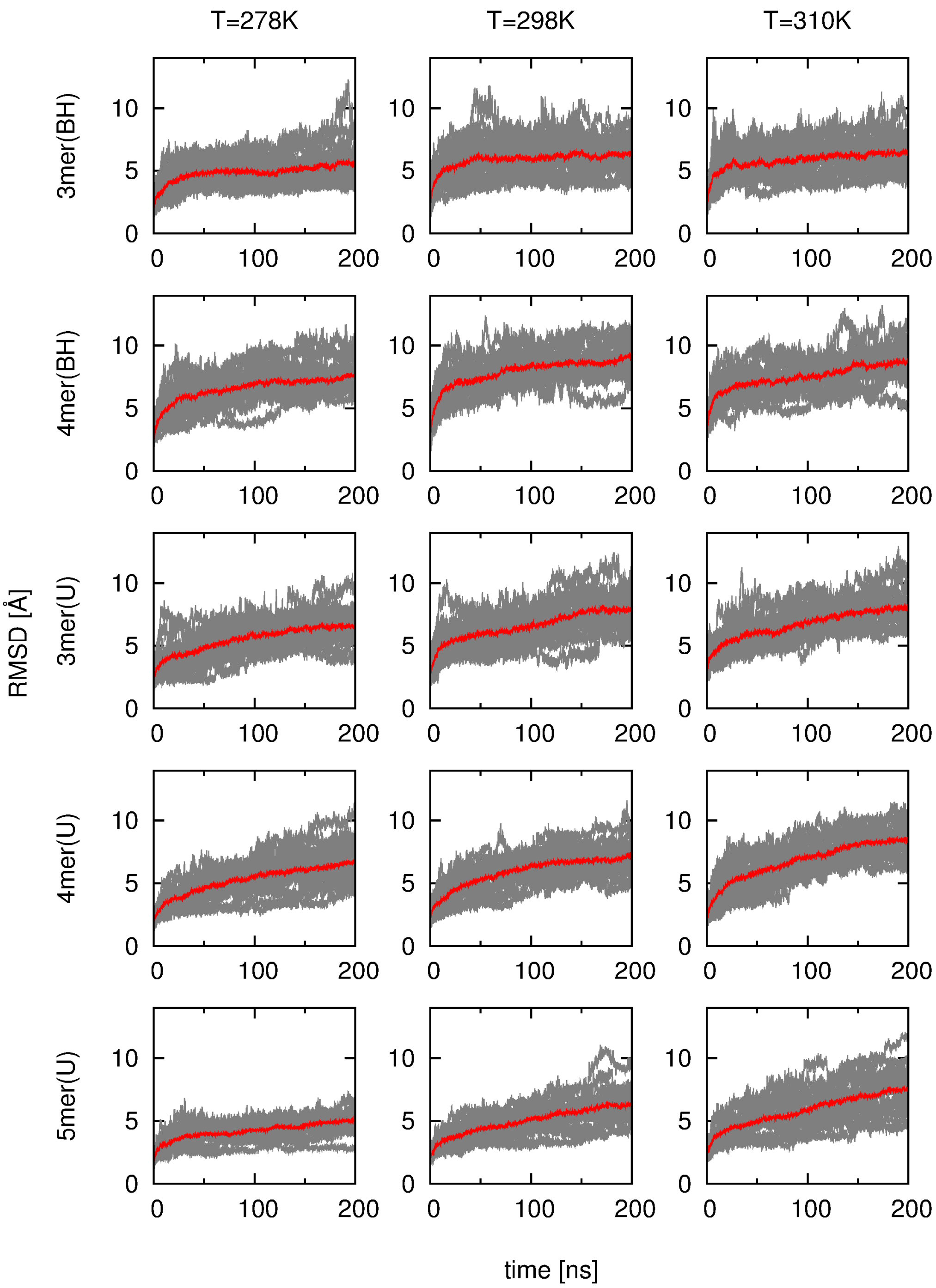

Supplementary Figure 10. Root mean square deviation (RMSD) for five structures (trimer BH, tetramer BH, trimer U, tetramer U, pentamer U) over 20 independent runs (gray color) at three temperatures 278K(5C°), 298K(25C°) and 310K(37C°). Red colored line represents the average RMSD. Trimer U is obtained by removing a peptide from tetramer U. Evidently, RMSD figures reveal that trimer BH is more stable than trimer U, tetramer U or tetramer BH.

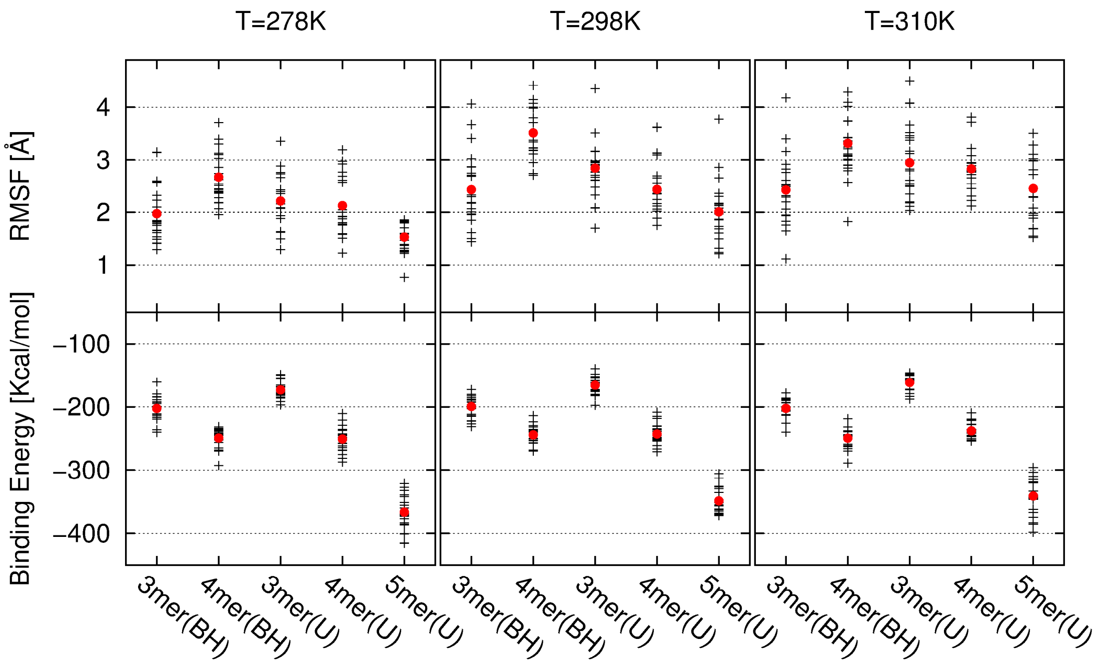

Supplementary Figure 11. Backbone atoms' RMSF(root mean square fluctuation) & binding energy including internal, electrostatic, van der Waals and solvation (GB, SA) energies. Independent twenty runs (cross symbol) and average values (red dots) are given for each structure. RMSF shows that trimer BH is more stable than tetramer BH, trimer U, and tetramer U, which explains why trimer BH + monomer can be preferred kinetically. Note that trimer BH has comparable RMSFs with tetramer U. Consequently, trimer BH can be stable even under all-atom simulations.

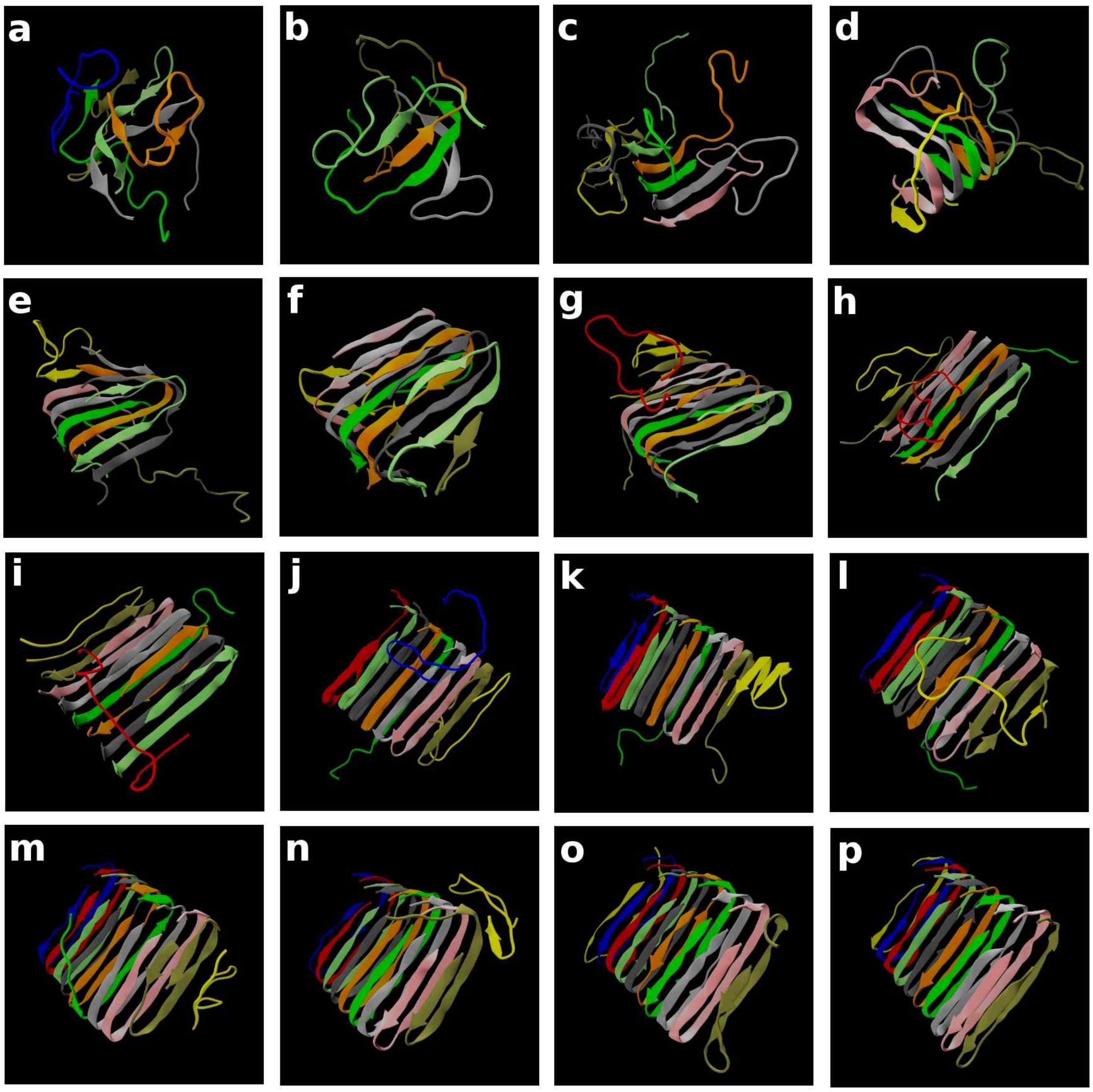

Supplementary Figure 12. Snapshots for a trajectory forming U-shape structure for NC=10. Snapshots are taken at (a) $t^* = t/\sigma(k_B T/m)^{1/2}$ = 4069, (b) 4996, (c) 5109, (d) 5444, (e) 6265, (f) 6646, (g) 11180, (h) 11243, (i) 11342, (j) 14992, (k) 18168, (l) 28610, (m) 28804, (n) 28846, (o) 30385, (p) 69298 (700 billion collisions). Snapshots from (a) to (i) show only large oligomer part without separated monomers for easy viewing. Snapshots from (j) to (p) display one oligomer with all 12 chains. We observe monomer additions to a template and conversion into a U-shape; for example red chain (g-j), blue chain (j-k).

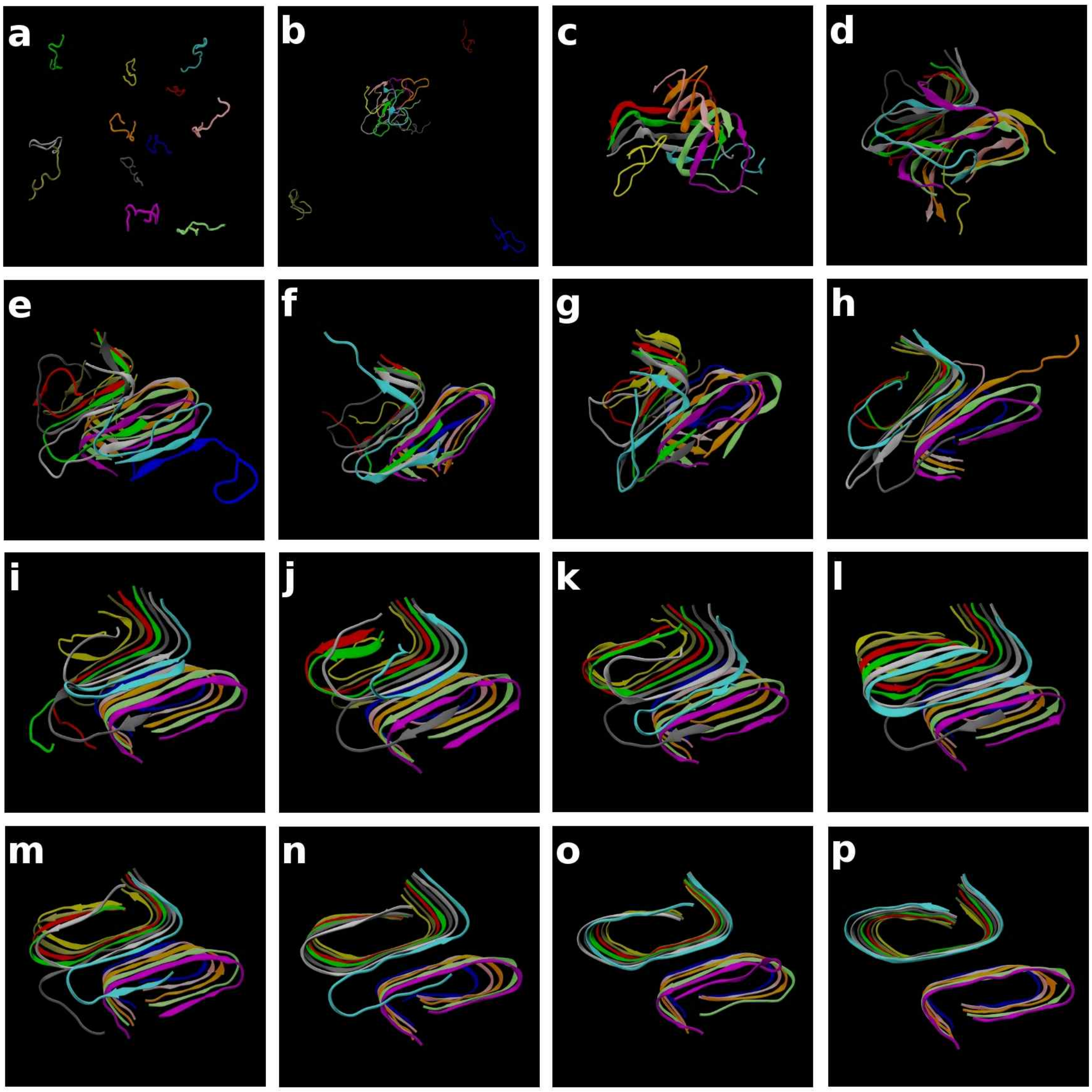

Supplementary Figure 13. Snapshots for a trajectory forming stacked U-shape structure for NC=12. Snapshots are taken at (a) $t^* = t/\sigma(k_BT/m)^{1/2} = 7$, (b) 1088, (c) 2523, (d) 5868, (e) 8444, (f) 14812, (g) 20954, (h) 22519, (i) 23799, (j) 24235, (k) 24923, (l) 33309, (m) 36018, (n) 36436, (o) 39015, (p) 50821. Chains of bottom right part of the oligomer are converted to U-shape first and the upper left chains are transformed to U-shape later.

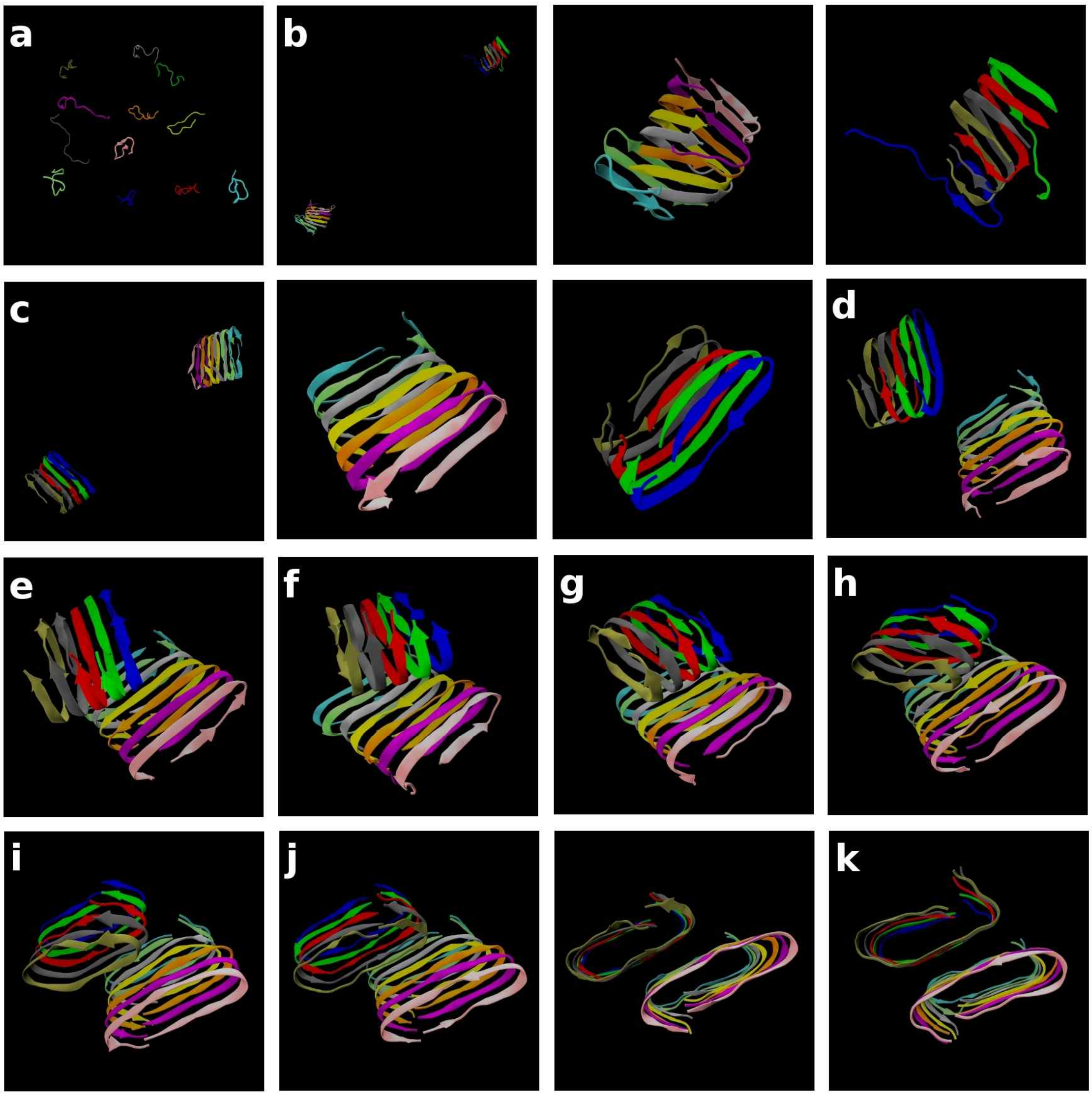

Supplementary Figure 14. Snapshots for a trajectory forming stacked U-shape structure for NC=12. Snapshots are taken at (a) t*= $t/\sigma(k_BT/m)^{1/2}$ = 7, (b) 4784, (c) 28390, (d) 59680, (e) 59694, (f) 59715, (g) 59737, (h) 59751, (i) 59765, (j) 59773, (k) 79446. Two oligomers are ordered separately at t*=28390 (c). After the lapse of long time, two ordered U-shape oligomers are merged at t*=59694 (e) and adjusted to form a stable stacked structure at t*=59773 (j). Although two pathways are quite different, the final two structures (Supplementary Fig.13p and Supplementary Fig.14k) look similar to each other. However, these two stacked quaternary structures are quite different from 2LMO if we look at the detailed structures of side-chain positions shown in Supplementary Fig.15 since these U-shape structures are out-of-registered and one of them is hybrid fibrillar.

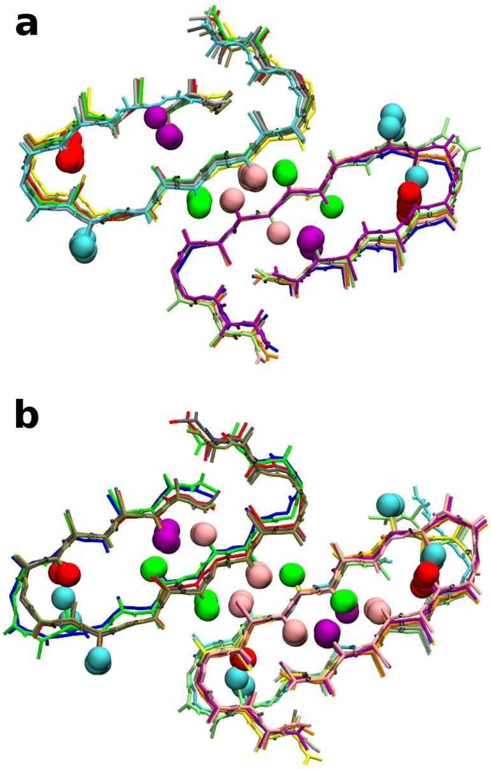

Supplementary Figure 15. The detailed structures having positions of side-chains for the two stacked fibrillar structures. Figures (a) and (b) are corresponding to Supplementary Fig.13 and 14 respectively. Side-chain spheres of F19(purple), D23(red), K28(cyan), I32(green) and L34(pink) are shown with neglecting other side-chain spheres for easy viewing. U-shape structures are not well ordered if we compare the detailed figures of Fig.2. They are out-of-registered and bottom part of figure (b) is hybrid. Formation of the salt-bridge and positions of hydrophobic residues are heterogeneous.

Amino Acids	DRC$_\alpha$(Å)	DRNH(Å)	DRCO(Å)
**R**	4.179	4.800	4.880
**N**	2.522	3.250	3.380
**D**	2.502	3.310	3.320
**Q**	3.135	3.820	3.940
**E**	3.137	3.830	3.940
**H**	3.163	3.720	3.850
**K**	3.515	4.180	4.270
**P**	1.926	1.851	2.995
**S**	1.967	2.790	2.890
**T**	1.981	2.770	2.940
**A**	1.600	2.503	2.573
**C**	2.339	3.100	3.250
**I**	2.308	3.030	3.250
**L**	2.621	3.340	3.510
**M**	3.198	3.880	4.000
**F**	3.396	3.890	4.040
**W**	3.881	4.380	4.510
**Y**	3.843	4.230	4.390
**V**	2.002	2.840	2.920

Supplementary Table 1. Parameters for covalent bond distance of Cα to side-chain sphere (DRCα), pseudo-bond distances of NH united sphere to side-chain sphere (DRNH) and of CO united sphere to side-chain sphere (DRCO).

Amino Acids	R(i) to Cα(i-1)	R(i) to CO(i-1)	R(i) to NH(i+1)	R(i) to Cα(i+1)	CO(i-1) to R(i+1)
**R**	5.395	4.557	4.386	5.211	4.594
**N**	4.423	3.429	3.380	4.464	4.397
**D**	4.584	3.586	3.245	4.342	4.482
**Q**	4.903	3.932	3.793	4.826	4.651
**E**	4.936	3.973	3.797	4.843	4.635
**H**	4.472	3.650	3.598	4.490	4.557
**K**	5.061	4.154	3.960	4.884	4.601
**P**	3.857	3.123	3.162	4.547	4.410
**S**	4.375	3.199	2.986	4.233	4.574
**T**	4.515	3.340	3.180	4.465	4.616
**A**	4.542	3.228	2.914	4.282	4.637
**C**	4.371	3.353	3.173	4.297	4.518
**I**	4.754	3.622	3.511	4.744	4.636
**L**	4.759	3.768	3.563	4.672	4.649
**M**	4.987	4.006	3.827	4.822	4.663
**F**	4.451	3.734	3.715	4.490	4.666
**W**	4.844	4.203	3.891	4.577	4.618
**Y**	4.523	3.918	3.963	4.594	4.668
**V**	4.657	3.468	3.288	4.542	4.630

Supplementary Table 2. The minimum distances between neighboring spheres not having covalent or pseudo-bonds. Distances of a side-chain sphere of i-th residue to a Cα sphere of i-1th residue (R(i) to Cα(i-1)), of a side-chain sphere of i-th residue to a CO sphere of i-1th residue (R(i) to CO(i-1)), of a side-chain sphere of i-th residue to a NH sphere of i+1 sphere (R(i) to NH(i+1)), of a side-chain sphere of i-th residue to a Cα sphere of i+1th residue (R(i) to Cα(i+1)), of a CO sphere of i-1th residue to a side-chain sphere of i+1th residue (CO(i-1) to R(i+1)). The minimum distance between CO(i-1) to R(i+1) should be 4.0Å if R(i+1) is PRO.

References

1. Cheon, M., Hall, C.K. & Chang, I. Structural Conversion of A beta(17-42) Peptides from Disordered Oligomers to U-Shape Protofilaments via Multiple Kinetic Pathways. *Plos Computational Biology* **11**(2015).
2. Govaerts, C., Wille, H., Prusiner, S.B. & Cohen, F.E. Evidence for assembly of prions with left-handed beta 3-helices into trimers. *Proceedings of the National Academy of Sciences of the United States of America* **101**, 8342-8347 (2004).
3. Van Melckebeke, H. et al. Atomic-Resolution Three-Dimensional Structure of HET-s(218-289) Amyloid Fibrils by Solid-State NMR Spectroscopy. *Journal of the American Chemical Society* **132**, 13765-13775 (2010).
